# Immune-Related lncRNA Risk Signatures Predict Survival of IDH Wild-Type and MGMT Promoter Unmethylated Glioblastoma

**DOI:** 10.1155/2020/1971284

**Published:** 2020-08-11

**Authors:** Xiaozhi Li, Yutong Meng

**Affiliations:** ^1^Department of Neurosurgery, Shengjing Hospital of China Medical University, Shenyang, China; ^2^Department of Stomatology, Shengjing Hospital of China Medical University, Shenyang, China

## Abstract

**Introduction:**

Glioblastoma is the most malignant grade of glioma, and it is also the most common primary tumor in the brain. Immunotherapy is a kind of precise tumor treatment. However, there are limited studies about immune-related lncRNA. This study is aimed at analyzing immune-related lncRNAs in glioblastoma and screening out prognostic factors, providing new potential targets for glioblastoma immunology research. *Material and Methods*. Gene expression data and clinical data of IDH wild-type with MGMT promoter unmethylated glioblastoma were acquired from the TCGA and CGGA databases. Immune-related lncRNAs were identified with the help of data from the InnateDB database. Immune prognostic factors were recognized by Cox regression analysis. GSEA analysis pursued their potential functions.

**Results:**

We found 318 immune-related lncRNAs. Among them, there were 137 immune-related lncRNAs that were upregulated and 181 that were downregulated. 15 prognostic lncRNAs were identified by Cox regression, and a total of 6 molecules were included in the following risk scoring model. GSEA showed that these lncRNAs participated in functions such as protein digestion and absorption and the PPAR signaling pathway.

**Conclusion:**

There are limited studies about immune regulation mechanisms of lncRNA in IDH wild-type with MGMT promoter unmethylated glioblastoma. The identified immune-related lncRNAs in glioblastoma might contribute new targets and research directions for immunological molecular studies of glioblastoma.

## 1. Introduction

Glioblastoma is the most malignant grade of glioma, and it is also the most common primary tumor in the brain. Despite the existence of neurosurgery, radiotherapy, chemotherapy, and other treatments, the median survival of glioblastoma patients is about 12 to 15 months [1, 2]. Moreover, the 5-year survival rate of glioblastoma is no more than 10% [3, 4]. Immunotherapy is a kind of precise tumor treatment, and it is one of the future development directions of cancer treatment. The discovery and application of immunological checkpoints, including PD-1 and CTLA4, provide new directions for the treatment of tumors [5–7]. However, although the anti-PD-1 drug pembrolizumab produced excellent antitumor effects and tolerance in the treatment of melanoma [8], studies have shown that the effect of this drug is far from satisfactory in the treatment of glioblastoma [9–11]. In addition, the immunological mechanisms of glioblastoma are not fully understood. In 2016, WHO conducted a molecular classification for glioma, pointing out the heterogeneity of glioma in various molecular states including IDH mutation status and MGMT promoter methylation status. Meanwhile, the molecular basis and prognosis of glioma patients with different IDH mutation status and MGMT promoter methylation status are significantly different [12, 13].

Long-chain noncoding RNAs (lncRNAs) take part in varieties of biological processes such as cell growth, differentiation, posttranscriptional regulation, inflammatory pathology, epigenetic regulation, and subcellular transport [14].

In recent years, some studies on lncRNA related to tumor immunity are being carried out [15–17]. The purpose of this study is to analyze immune-related lncRNA molecules of IDH wild-type and MGMT promoter unmethylated glioblastoma and screen out prognostic factors, providing new potential targets for glioblastoma immunology research.

## 2. Material and Methods

### 2.1. Data Source

Gene expression data and clinical data of IDH wild-type and MGMT promoter unmethylated glioblastoma were obtained from the public CGGA (http://www.cgga.org.cn/) and TCGA (https://cancergenome. https://nih.gov/) databases. CGGA contained two glioma data sets, namely, mRNAseq_325 and mRNAseq_693. TCGA and CGGA gene expression data were corrected using the “limma” package of R software. Because TCGA data contained gene expression of normal brain tissue, this study used TCGA data for differentially expressed gene screening, TCGA and CGGA (mRNAseq_325) data to construct a risk scoring model, and CGGA (mRNAseq_693) for model verification. Differentially expressed lncRNAs of the TCGA database were identified by the “edgeR” package of R software. ∣log2FC | >1 and false discovery rate < 0.05 were considered statistically different.

### 2.2. Immune-Related lncRNA Recognition

The list of immunomodulatory-related genes was obtained from the InnateDB website (https://www.innatedb.com). Additionally, when *P* < 0.05 and the correlation coefficient > 0.4, the correlation between RNAs was considered significant.

### 2.3. Cox Regression and Kaplan-Meier Analysis

Differentially expressed immune-related lncRNAs and clinical data were merged. Univariate Cox regression analysis was applied to find the prognostic ones. Furthermore, the risk score was established by stepwise multivariate Cox regression analysis. The Kaplan-Meier survival curve in high-risk and low-risk groups of glioblastoma was constructed based on the median risk score. The area under the curve (AUC) of the receiver operating characteristic (ROC) curve was applied to assess the clinical prognosis ability of the risk scoring model for glioblastoma patients.

### 2.4. Gene Set Enrichment Analysis (GSEA)

The R software “clusterProfiler” package was used to perform Gene Ontology (GO) enrichment analysis and Kyoto Encyclopedia of Genes and Genomes (KEGG) enrichment analysis between genes in low-risk and high-risk cohorts in order to pursue their possible functions.

### 2.5. Statistical Analysis

All statistical analyses in this study were performed with R software 4.0.0. Comparison between groups for abnormal distributed variables was performed by Mann–Whitney *U* test. Correlation between RNAs was assessed by Pearson correlation test.

## 3. Results

### 3.1. Differentially Expressed lncRNAs in IDH Wild-Type with MGMT Promoter Unmethylated Glioblastoma

A total of 5 normal brain tissue expression samples and 66 IDH wild-type with MGMT promoter unmethylated glioblastoma samples were included in the TCGA database. The diagnostic age range of glioma patients was 24-89 years old. At the end point of last follow-up, 23 patients survived and 43 died. By the analysis of the “edgeR” package, 318 differentially expressed lncRNAs were identified. There were 137 upregulated and 181 downregulated lncRNAs.

**Figure 1:**
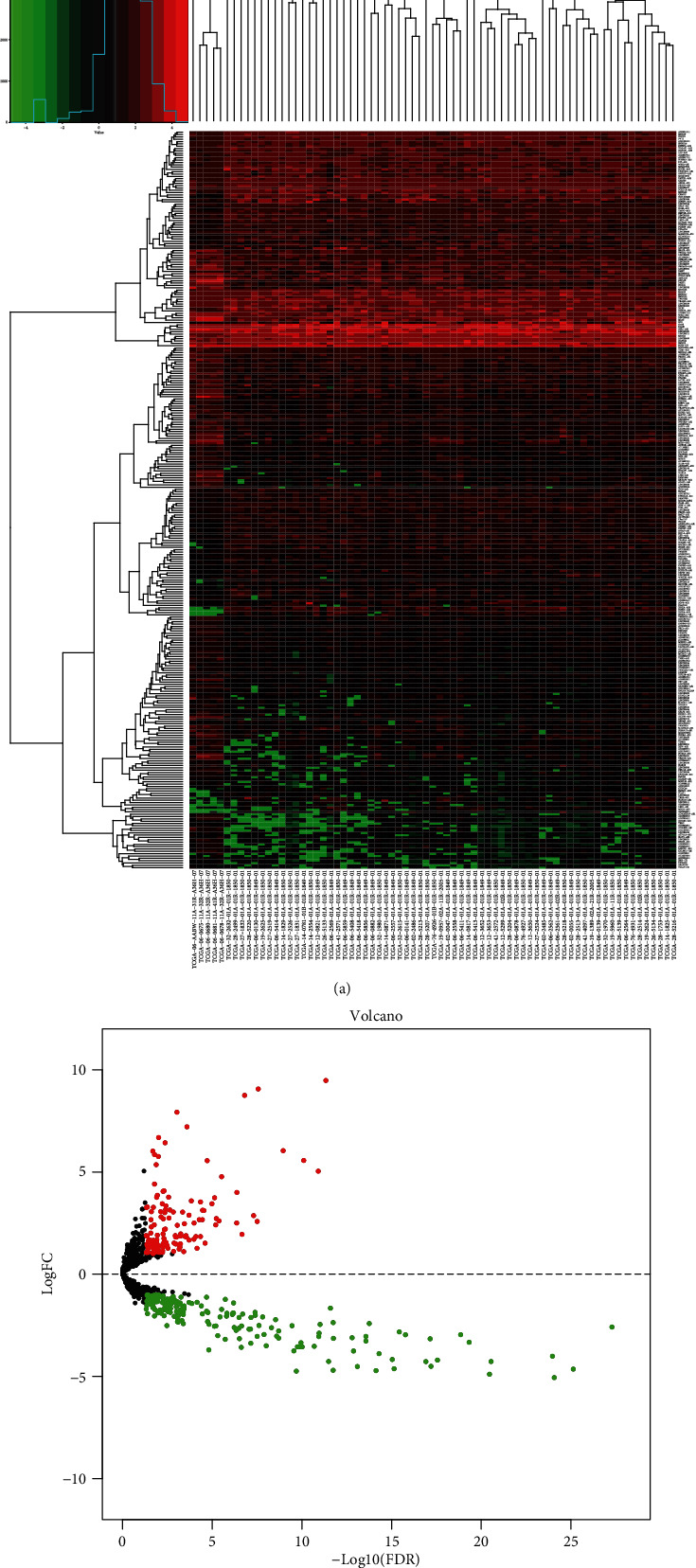
Differential expression immune-related lncRNAs. (a) Heat map of immune-related lncRNAs. Red blocks represent upregulation whereas green blocks represent downregulation. (b) Volcano plot of immune-related lncRNAs. Red points represent upregulation whereas green points represent downregulation.

A list of immune-associated genes was acquired from the InnateDB website. A total of 1697 immunomodulatory-related genes were extracted. Interestingly, the 318 differentially expressed lncRNAs previously found were all immune-related genes according to the correlation coefficients between molecules. The corresponding heat map and volcano plot are displayed in Figure 1.

### 3.2. Univariate and Multivariate Cox Regression

**Figure 2:**
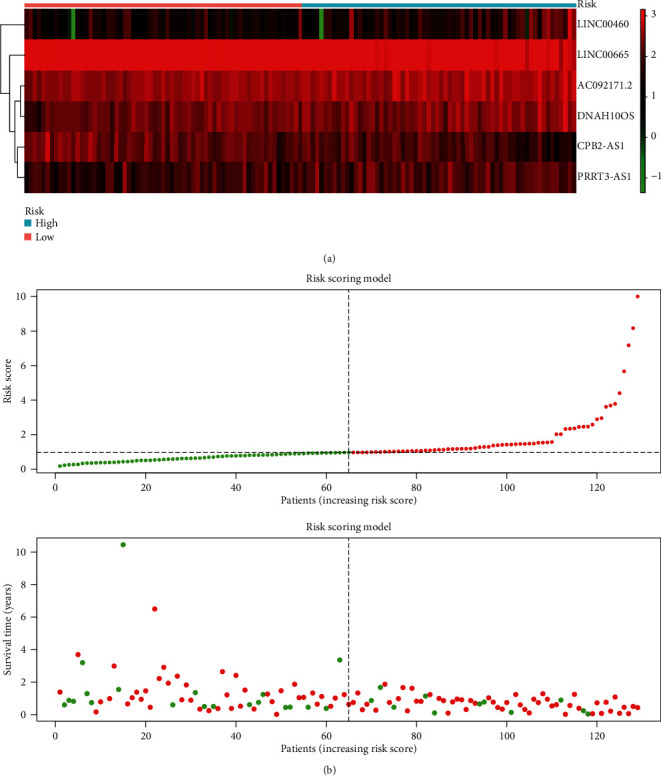
Prognostic immune lncRNAs. (a) Heat map of prognostic immune lncRNAs. (b) The relationship between risk scores and overall survival.

Using TCGA and CGGA (mRNAseq_325) as the training cohort, 15 immune-related prognostic lncRNAs were identified by univariate Cox regression, including LINC00461, LINC00511, CPB2-AS1, AC092171.2, LINC00665, ARHGAP31-AS1, AC006449.1, TRAPPC12-AS1, AC144831.1, TRAM2-AS1, LINC00460, PRRT3-AS1, CACNA1C-AS1, RNF219-AS1, and DNAH10OS. Then, the prognostic model was built by stepwise multivariate Cox regression. A total of 6 lncRNAs were included in the risk scoring model: risk score = −0.344∗expression_CPB2−AS1_ + 0.520∗expression_AC092171.2_ − 0.341∗expression_LINC00665_ + 0.166∗expression_LINC00460_ + 0.202∗expression_PRRT3−AS1_ + 0.298∗expression_DNAH10OS_. Among them, 2 lncRNAs (CPB2-AS1 and AC092171.2) were independent prognostic factors for IDH wild-type with MGMT promoter unmethylated glioblastoma. The results of the Cox regression are displayed in Table 1. The heat map of all molecules involved in the risk score is shown in Figure 2(a). Besides, the relationship between risk scores and overall survival is shown in Figure 2(b).

**Figure 3:**
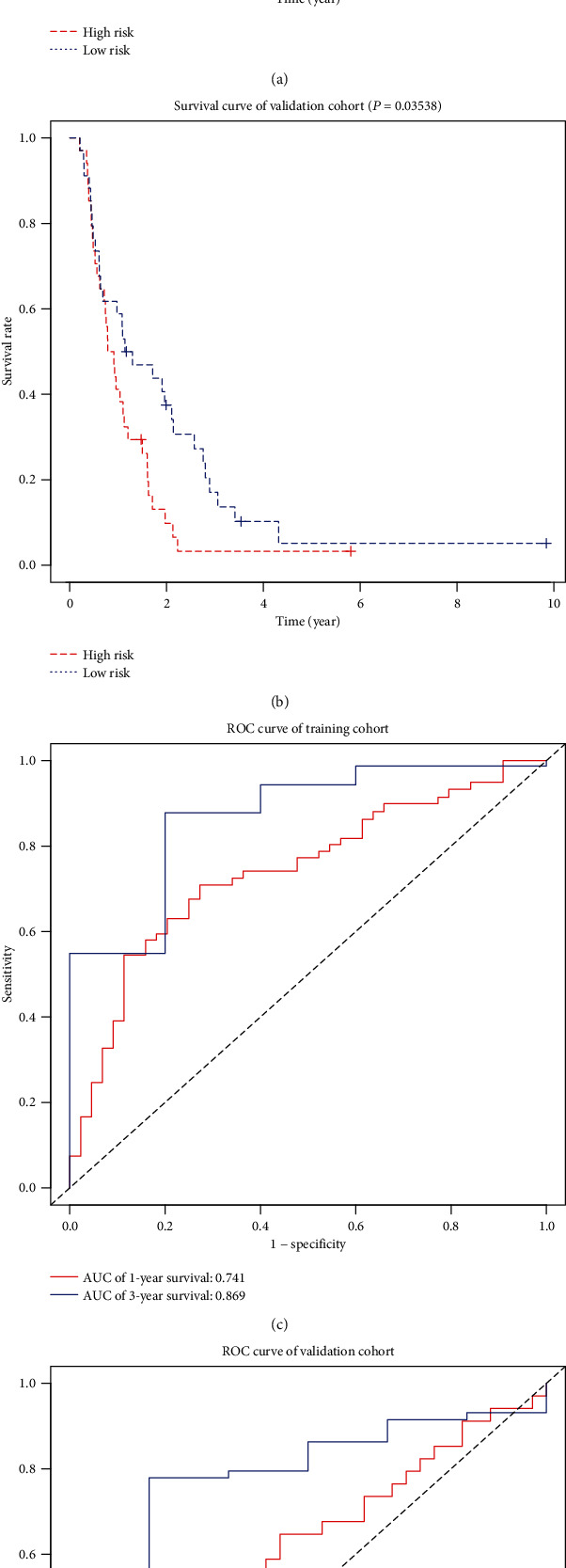
Testing of the construction of the risk scoring model. (a) Kaplan-Meier survival curve of the training cohort. (b) Kaplan-Meier survival curve of the validation cohort. (c) The ROC curve of the training cohort. (d) The ROC curve of the validation cohort.

In order to verify the ability of predicting overall survival of glioma patients, glioblastoma samples were divided into low-risk and high-risk groups based on the median value of the risk score. Through the Kaplan-Meier survival curves, overall survival of low-risk patients was much higher than that of their high-risk counterparts (training cohort *P* < 0.001; validation cohort *P* = 0.035), displayed in Figure 3(a). In addition, ROC curves of the training group and the verification group are displayed in Figure 3(b). The AUC values of 1-year and 3-year ROC curves were 0.741 and 0.869, respectively, in the training cohort, while in the verification group, the AUC values of the 1-year and 3-year ROC curves were 0.554 and 0.728, respectively. The ROC curves showed that the risk score had a good predictive ability for the prognosis of glioblastoma.

**Figure 4:**
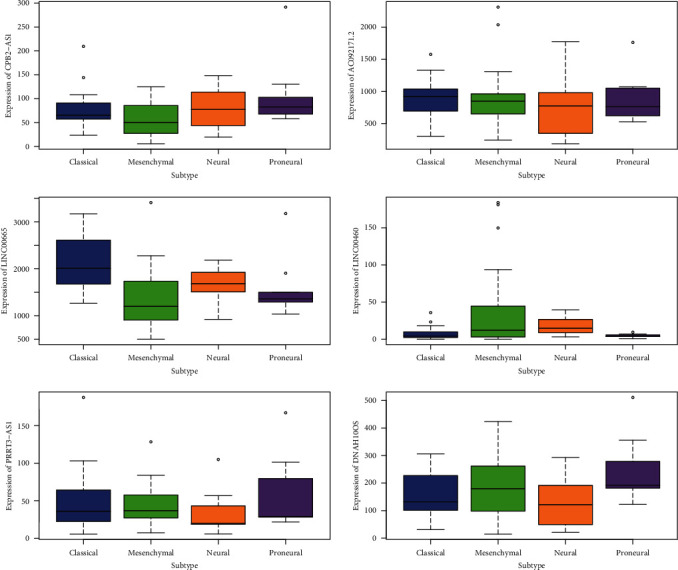
6 lncRNA (CPB2-AS1, AC092171.2, LINC00665 and LINC00460, PRRT3-AS1, and DNAH10OS) expression in subtypes of glioblastoma.

In addition, the expression of CPB2-AS1, AC092171.2, LINC00665 and LINC00460, PRRT3-AS1, and DNAH10OS in each subtype of glioblastoma is shown in Figure 4.

### 3.3. GSEA

**Figure 5:**
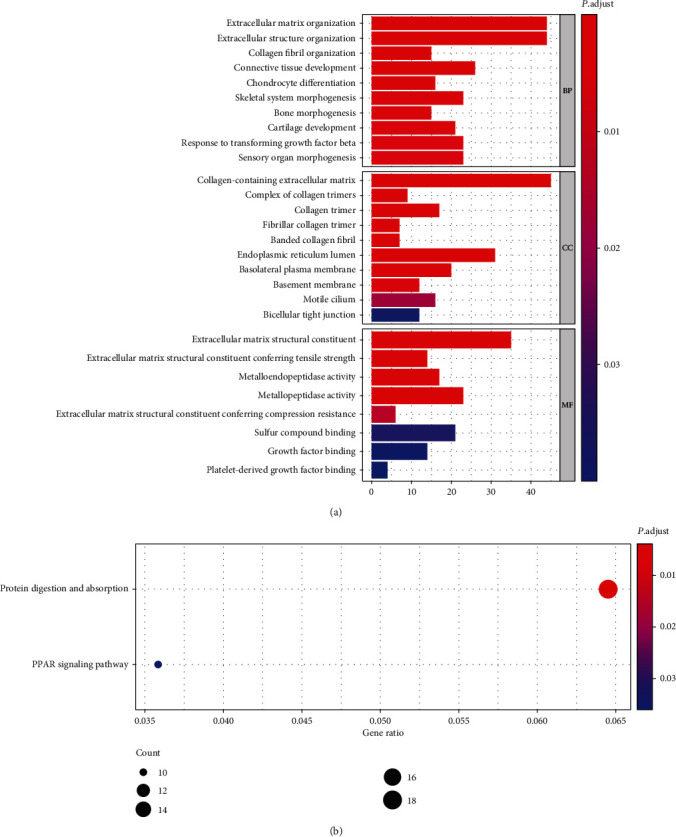
GSEA: (a) GO analysis results; (b) KEGG analysis results.

We performed the functional enrichment analysis on the differentially expressed lncRNAs (Figure 5). The GO enrichment analysis suggested that the differential expressed lncRNAs were enriched in items such as extracellular matrix organization, collagen-containing extracellular matrix, and extracellular matrix structure constituent. KEGG analysis showed that these lncRNAs were involved in pathways such as protein digestion and absorption and PPAR signaling pathway.

## 4. Discussion

The treatment of glioblastoma is challenging. Immune checkpoints can modulate the magnitude and quality of T cell responses, and tumors use these mechanisms to inactivate T cells in an immunosuppressive microenvironment [18]. The production of the immunosuppressive microenvironment (TME) makes immunotherapy effects in patients with glioblastoma unsatisfactory [19]. Checkpoint inhibitors such as anti-PD-1, anti-PDL1, and anti-CTLA4 have been used in many tumors. In a mouse model of glioblastoma, anti-PD-1 therapy combined with TMZ can eradicate glioblastoma [20]. However, single checkpoint inhibitors have little effect on the treatment of most GBM patients [21]. This may be due to the specificity of glioblastoma immunization and the heterogeneity of glioblastoma itself, so more in-depth studies are needed to identify new and effective checkpoints. Glioblastoma immune-microenvironment presents an antitumor immune response. Though little is known about immunosuppression, regulation of the immune system is crucial for the treatment of glioblastoma [22].

In this study, differentially expressed lncRNAs of the IDH wild-type with MGMT promoter unmethylated glioblastoma were analyzed, and Cox regression was used to construct a prognostic model for glioma patients. Kaplan-Meier curves and ROC curves suggested that the risk scoring model based on the 6 lncRNAs (CPB2-AS1, AC092171.2, LINC00665 and LINC00460, PRRT3-AS1, and DNAH10OS) had a strong predictive ability for glioblastoma patients.

There have been some lncRNA analyses of glioma. For example, Li et al. has applied immune lncRNA analysis for pancancer characterization based on the TCGA database [23]. Moreover, we have also previously conducted studies on a lncRNA immune study of low-grade gliomas based on the TCGA database [24]. Zhou et al. analyzed all the lncRNAs of glioblastomas based on the TCGA database [25]. This study, due to the strong difference in molecular bases and prognosis of glioblastoma patients with different IDH mutation with MGMT promoter methylation status, conducted a multidatabase analysis (TCGA and CGGA databases) to immune-related lncRNA analysis of the IDH wild-type and MGMT promoter unmethylated glioblastoma, to provide targets for immunological studies of glioblastoma.

Through the correlation analysis, 6 immune-related lncRNAs were identified, some of which have been reported in the previous studies. For example, LINC00665 is downregulated in glioma and mediates STAU1-mediated MTF1 and YY2 stability, affecting malignant biological behavior of glioma [26]. Besides, LINC00460 is upregulated in glioma. LINC00460 can promote the proliferation, migration, and invasion of glioma cells by negatively regulating miR-320a [27]. In prostate cancer, PRRT3-AS1 silencing can block the mTOR signaling pathway, promote tumor cell autophagy, and inhibit tumor cell proliferation [28]. However, PRRT3-AS1 has not been reported involving immune regulation of glioma.

There are limitations in the study. First of all, this study is a retrospective study based on high-throughput sequencing data and requires the verification of many further basic experiments. Secondly, this study focuses on the IDH wild-type with MGMT promoter unmethylated glioblastoma. A more detailed comparison of molecular subtypes of glioblastoma may require larger sequencing data to obtain better results.

## 5. Conclusion

This study identified a total of 318 immune-related lncRNAs in IDH wild-type with MGMT promoter unmethylated glioblastoma. Univariate Cox regression reveals 15 prognosis-related lncRNAs. Six lncRNAs were considered to be independent prognostic risk factors for glioblastoma. GO and KEGG analyses suggested that the lncRNAs may involve signaling pathways such as extracellular matrix organization, collagen-containing extracellular matrix, extracellular matrix structure constituent, protein digestion and absorption, and PPAR signaling pathway. This study is aimed at contributing new targets and research directions for immunological molecular studies of glioblastoma.

## Figures and Tables

**Table 1:** The Cox regression results of immune-related lncRNAs. Univariate analysis and multivariate cox regression of immune-associated lncRNAs.

lncRNA	Univariate analysis		Multivariate analysis	
	HR (95% CI)	*P*	HR (95% CI)	*P*
CPB2-AS1	0.599 (0.459-0.781)	0.000	0.709 (0.547-0.918)	0.009
AC092171.2	1.587 (1.076-2.340)	0.020	1.681 (1.074-2.634)	0.023
LINC00665	0.653 (0.462-0.922)	0.016	0.711 (0.489-1.033)	0.073
LINC00460	1.279 (1.088-1.504)	0.003	1.180 (0.987-1.411)	0.060
PRRT3-AS1	1.241 (1.006-1.531)	0.044	1.224 (0.954-1.569)	0.112
DNAH10OS	1.477 (1.102-1.980)	0.009	1.347 (0.966-1.876)	0.079

## Data Availability

The data in this study was available from the corresponding author on reasonable request.
